# Anti-Anginal Efficacy of Zibotentan in the Coronary Slow-Flow Phenomenon

**DOI:** 10.3390/jcm13051337

**Published:** 2024-02-27

**Authors:** Sivabaskari Pasupathy, Rosanna Tavella, Christopher Zeitz, Suzanne Edwards, Matthew Worthley, Margaret Arstall, John F. Beltrame

**Affiliations:** 1 School of Medicine, Faculty of Health Sciences, The University of Adelaide, Adelaide, SA 5000, Australia; sivabaskari.pasupathy@adelaide.edu.au (S.P.); rosanna.tavella@adelaide.edu.au (R.T.); christopher.zeitz@adelaide.edu.au (C.Z.); suzanne.edwards@adelaide.edu.au (S.E.); matthew.worthley@adelaide.edu.au (M.W.); margaret.arstall@adelaide.edu.au (M.A.); 2 Central Adelaide Local Health Network, Adelaide, SA 5000, Australia; 3 Basil Hetzel Institute for Translational Health Research, Adelaide, SA 5011, Australia; 4 Northern Adelaide Local Health Network, Adelaide, SA 5112, Australia

**Keywords:** coronary microvascular dysfunction, coronary slow-flow phenomenon, zibotentan

## Abstract

Background: Patients with coronary microvascular disorders often experience recurrent angina for which there are limited evidence-based therapies. These patients have been found to exhibit increased plasma levels of endothelin; thus, selective endothelin–A (Et-A) receptor blockers such as zibotentan may be an effective anti-anginal therapy in these patients. The study evaluated the impact of a 10 mg daily dose of zibotentan on spontaneous angina episodes in patients with the coronary slow-flow phenomenon who had refractory angina (i.e., experiencing angina at least three times/week despite current anti-anginal therapy). Methods: Using a randomized, double-blind, placebo-controlled, crossover trial design with 4-week treatment periods, 18 patients (63.2 ± 9.9 years, 33% females) were recruited. The primary endpoint was angina frequency as measured by an angina diary, with secondary endpoints including nitrate consumption, angina duration/severity and the Seattle Angina Questionnaire (SAQ) domains. Results: During the 4 weeks of therapy, angina frequency significantly improved with zibotentan therapy (placebo 41.4 (58.5) vs. zibotentan 29.2 (31.6), *p* < 0.05), and sublingual nitrate consumption significantly reduced (placebo 11.8 (15.2) vs. zibotentan 8.8 (12.9), *p* < 0.05. Conclusions: Zibotentan improved the frequency of spontaneous angina episodes and reduced sublingual nitrate consumption in patients unresponsive to standard anti-anginal therapy.

## 1. Introduction

Coronary microvascular dysfunction (CMD) represents a subset of cardiovascular pathologies characterized by structural and/or functional anomalies within the coronary microcirculation [1]. Diverging from conventional atherosclerotic large-vessel coronary artery disease (CAD), CMD predominantly impacts the intricately organized network of microvessels responsible for myocardial perfusion. Although CMD may co-exist with other cardiac disorders (e.g., CAD and myocardial disorders) [1], several primary coronary microvascular disorders have been clinically described and attributed to CMD [2].

One subtype of primary coronary microvascular disorders is the coronary slow-flow phenomenon (CSFP). This angiographic entity is characterized by the slow passage of angiographic contrast in the absence of obstructive CAD and is thus attributed to an increased coronary microvascular resistance. With a prevalence of 1–7% in patients undergoing diagnostic angiography for the investigation of chest pain [3,4], it is well recognised by clinicians undertaking invasive coronary angiography. The CSFP has been clinically characterized by studies demonstrating novel features including frequent unstable angina presentations [5], an increased resting coronary microvascular resistance [6], inflammation associated with the unstable angina presentations [7], and a predilection to recalcitrant angina symptoms [8]. This latter clinical characteristic has prompted several studies evaluating novel therapies for the CSFP, including mibefradil [9], nebivolol [10], statins [11], and nicorandil [12].

Clinical experience indicates the limited efficacy of standard antianginal medical therapy in the chronic management of the CSFP. Vasodilators such as dipyridamole and adenosine, which affect the microvessels, have been shown to improve coronary flow in CSFP patients, whereas those targeting larger epicardial coronary arteries, such as GTN, have failed to normalize coronary flow in the catheterization lab setting [4]. In a seminal trial involving 20 CSFP patients, the administration of 100 mg of mibefradil daily resulted in acute improvements in coronary blood flow, demonstrating a 56% reduction in spontaneous angina episodes, a 74% decrease in prolonged angina episodes (>20 min), and an improvement in quality of life based on the health outcome study Short Form-36 (SF-36) [9]. However, mibefradil was withdrawn from general therapeutic availability due to its inhibition of cytochrome P450 3A4, precipitating unwarranted drug interactions [13]. Smoking cessation is a nonpharmacologic intervention in the treatment of CSFP patients due to its association with the condition [14]. Despite these therapeutic approaches, there are currently no recognized effective therapies for this coronary microvascular disorder.

An innovative therapeutic approach in the treatment of CSFP is to consider an endothelin-receptor blocker as an anti-anginal agent. Endothelin-1 is a potent endogenous vasoconstrictor peptide that stimulates both Endothelin-A (Et-A) and Endothelin-B (Et-B) receptors [15]. The stimulation of Et-A receptors produces vasoconstriction, whereas the Et-B receptor stimulation induces the release of counter-regulatory vasodilators (i.e., nitric oxide) [16,17]. In the context of CSFP, several observations indicate the involvement of Endothelin-1 (Et-1) in the pathogenesis of this coronary microvascular disorder: (a) intracoronary ET-1 administration in canine [18] and rabbit models [18,19] replicates the angiographic features of the CSFP; (b) patients with CSFP exhibit elevated systemic ET-1 levels that further increase during exercise [20,21]; (c) coronary sinus Et-1 levels are increased in the CSFP and further escalate with rapid atrial pacing [22]; (d) intravenous ET-1 infusion into healthy individuals induces a reduction in coronary sinus oxygen saturation [23], mirroring clinical observations in patients with CSFP [6]; and (e) isolated subcutaneous microvessels of CSFP patients demonstrate a selective hyper-responsiveness to ET-1 [24]. In addition, both Et-A and Et-B receptors are found within the human coronary microvasculature, implying that they play a key role in the regulation of coronary microvessels [25]. Accordingly, Et-1 blockade may be an effective therapy in the management of recurrent angina experienced by these patients.

Initially designed for the treatment of prostate cancer [26], zibotentan is an ET-1 blocker that selectively inhibits the vasoconstricting Et-A receptor but not the Et-B receptor. It is currently being investigated as a potential therapeutic option for chronic kidney disease [27]. This pilot study assesses the anti-anginal impact of targeted Et-A receptor blockade with zibotentan in patients with CSFP. Specifically, the primary objective of this study is to evaluate the effect of a 10 mg dose of zibotentan once daily on angina frequency in patients with CSFP who experience angina at least three times per week. Secondary objectives include assessing the impact of zibotentan on sublingual nitrate consumption, the frequency of prolonged angina episodes (>20 min), physical limitations, as measured by the Seattle Angina Questionnaire (SAQ), quality of life, as assessed by SAQ, physical health, scored by the Short Form-36 (SF-36) physical health summary score, and mental health, scored by the SF-36 mental health summary score, in patients with symptomatic CSFP. In addition, patient compliance with taking medication and adverse events will be recorded.

## 2. Methods

To achieve the above objectives, a single-centre, randomized, double-blind, placebo-controlled, crossover trial design was utilized. The study protocol was approved by the Central Adelaide Local Health Network ethics committee, Adelaide, Australia and is registered with Australian New Zealand Clinical Trials Registry (ACTRN12618000021279).

### 2.1. Study Cohort

Patients with stable angina patterns and non-obstructive CAD were screened and recruited if they satisfied the inclusion/exclusion criteria outlined below. Inclusion criteria were as follows: (i) angiographically confirmed CSFP (i.e., TIMI-2 flow in the absence of obstructive CAD [<50% stenosis]) and (ii) refractory angina (i.e., ≥3 episodes/week, despite maintenance treatment including long-acting nitrates, beta-blockers, and/or calcium channel blockers). Exclusion criteria comprised the following: (i) admission for acute coronary syndrome within the past month; (ii) secondary causes contributing to the CSFP, such as the no-reflow phenomenon and myocarditis; (iii) secondary causes of angina, including clinically significant conditions such as anemia (hemoglobin < 100 g/dL), uncontrolled atrial fibrillation (i.e., ventricular response rate > 108 bpm), and hemodynamically significant aortic stenosis (estimated mean aortic valve gradient ≥40 mmHg); (iv) contraindications to study treatment; (v) women of childbearing potential or those with known pregnancy or breastfeeding; (vi) patients with pregnant partners; (vii) individuals with a history of cancer, malignancy, or substance abuse; (viii) use of an endothelin receptor antagonist within 3 months prior to the study’s initiation; (ix) abnormalities in liver function tests.

### 2.2. Study Protocol and Treatments

Investigational products, encompassing both the study drug and the placebo, were identical in form and were manufactured and sponsored by AstraZeneca Pty Limited, Cambridge, UK. The randomization process utilized a computer-generated algorithm, and the sequence was exclusively known to the hospital clinical study pharmacist (who had no patient or clinician contact) until the completion of data analysis. Eligible patients, upon providing informed consent, underwent initial assessments including clinical examination, blood-pressure and heart-rate monitoring, a 12-lead electrocardiogram, a medical history review, and the completion of the SAQ and SF-36 questionnaires. Following this, patients maintained a two-week angina diary to evaluate baseline chest pain frequency. Only those experiencing angina at least three times per week proceeded to randomization and initiated study medication.

Participants underwent a 4-week treatment followed by a 2-week washout before crossing over to the second treatment phase for an additional 4 weeks. They attended the trial clinic at the initiation and conclusion of each phase. Additionally, they were contacted via phone during the treatment phases to monitor any potential adverse reactions to the drug. Participants were given angina diaries at each visit, with instructions to consistently document the occurrence, severity (on a scale of 1–10), and duration (including episodes lasting >20 min) of, as well as sublingual nitrate (GTN) consumption during, angina episodes. During each visit, a case report form was completed, recording clinical observations such as heart rate, blood pressure, ECG, safety blood results, and questionnaire responses. Any modifications to trial medication, including compliance and adjustments to other medications, were documented in the case report form. Adverse events were documented based on participant-provided descriptions, prompted by an open question regarding changes in health status, medical conditions, or any adverse events experienced since their last study contact. Furthermore, participants were explicitly queried about the occurrence of peripheral edema, headaches, nasal congestion, anaemia, and light-headedness. If light-headedness was reported, and if participants were already on antihypertensive medication and not influencing angina, then medication was preferentially reduced. In other instances, the study treatment was reduced.

### 2.3. Study Endpoints

The primary endpoint was angina frequency as measured by a comprehensive angina diary. Secondary endpoints encompassed the consumption of GTN, the overall duration of angina episodes, and patient experiences utilizing the SAQ and the SF-36 domains.

### 2.4. Data Analysis

For this crossover trial, a linear mixed-effects model was used to assess the association between angina diary variables (angina frequency, prolonged angina episodes, etc.) and the fixed effects: month (phase 1 vs. phase 2), treatment group (X or Y), and study arm order (X then Y or Y then X). To adjust for repeated measurements over time, participant ID number was included as a random effect with a compound symmetry covariance structure. Linear mixed-effects models were used to assess the association between the SAQ outcome variables, SF-36 variables and the fixed effects: treatment group (X or Y) and study arm order (X then Y or Y then X). The normality of residuals and homoscedasticity were found to be upheld by inspection of a histogram of residuals and scatter plot of predicted values and residuals. A long format of the dataset was used to perform these linear mixed-effects models. The statistical software used was SAS On Demand for Academics (SAS Institute Inc. 2021), Version 9.4, Cary, NC, USA. A *p* value < 0.05 was considered statistically significant. The data analysis was performed in a blinded manner, without access to the randomization information. Following the completion of data analysis, the pharmacist unblinded the corresponding designation for zibotentan.

The study sample size was calculated based on the primary endpoint of total angina frequency. Using data from a previous study on mibefradil efficacy on CSFP [9], where total angina frequency during placebo therapy was 28 ± 31 episodes/month, a 56% reduction was observed with the active study drug. Employing a crossover design to detect a 50% change with zibotentan, 39 patients, accounting for dropouts, were calculated for 80% power at alpha = 0.05.

## 3. Results

**Figure 1:**
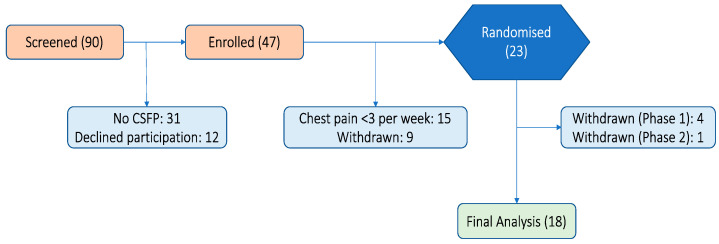
Consort diagram.

From June 2018 to April 2022, a total of 23 participants were randomised, and 5 individuals subsequently withdrew from the study before successfully completing the study protocol. Four subjects withdrew, all citing personal reasons such as travel commitments or unforeseen family emergencies—three before the administration of any study drug and one after completing Phase 1. The fifth participant withdrew from the study due to a non-cardiac hospital admission unrelated to their study participation (see Figure 1). The final analysis included 18 subjects (63.2 ± 9.9 years, 33% female). Risk factors included diabetes (11%), hypertension (56%), and dyslipidemia (56%). Baseline anti-anginal therapy included calcium channel blocker (77%), beta blocker (11%), long-acting nitrates (61%), and other anti-anginals (22%) (Table 1).

### 3.1. Primary and Secondary Endpoints

Compared to the placebo, the 4-week zibotentan therapy led to a 31% reduction in angina frequency and a concurrent 33% decrease in sublingual nitrate use, as recorded in the angina diary (Table 2). However, there were no significant differences observed in the frequency of prolonged angina episodes, angina severity, SAQ domains, or SF-36 summary scores between the placebo and zibotentan groups (Table 3). Medication compliance exceeded 95%, as assessed by pill count, and concomitant medications remained unchanged throughout the study. 

### 3.2. Tolerability and Safety Endpoints

The study drug was generally well-tolerated, with no serious drug-related adverse events. However, possible study-drug-related adverse reactions were observed in approximately 45% of participants. The study drug was not discontinued in any patient but in two participants, the study dose was halved. In another two patients, perindopril was halved due to low symptomatic blood pressure. Overall, there were no clinically significant changes in laboratory values, vital signs, electrocardiogram measurements, and physical examination between treatment arms (Table 4).

## 4. Discussion

This small pilot study demonstrates, for the first time, an anti-anginal benefit of selective Et-A blockade in patients with a coronary microvascular disorder. Specifically, 10 mg of zibotentan daily reduced spontaneous angina episodes in patients with CSFP experiencing refractory angina compared to conventional anti-anginal therapies. Consistent with this observation, there was a reduction in short-acting sublingual nitrate consumption. The importance of these findings lies in their clinical translation, considering the patient-related primary outcome of spontaneous episodes of angina.

The findings contrast with our previous study using a combined Et-A/Et-B receptor blocker (bosentan), where a slightly larger study cohort (n = 23) did not demonstrate an anti-anginal benefit, although there was a non-significant trend [28]. Thus, further studies should explore the utility of the selective Et-A blockade as an anti-anginal agent in patients with CMD. Moreover, whether these clinical benefits are only evident in patients with CSFP or can be extended to other coronary microvascular disorders also requires further investigation.

Despite the documented clinical benefits in the study’s primary endpoint (angina frequency recorded via an angina diary), the key secondary endpoints showed no significant differences. This included the SAQ angina frequency domain, which considers both the frequency of angina episodes and short-acting nitrate consumption. Two explanations may account for this apparent disparity. Firstly, and most importantly, is the small sample size, since the SAQ angina frequency domain did exhibit a 10-point difference between treatments, although this was not statistically significant (Table 3). A 10-point difference in SAQ scores was previously shown to be clinically significant [29]. Secondly, the angina diary was prospectively collected and contains more granular detail than the SAQ angina frequency domain, which requires the recollection of angina frequency and nitrate consumption over the preceding 4 weeks. Hence, with a smaller sample size and prospectively collected granular data, an anti-anginal benefit of zibotentan could be demonstrated using the angina diary.

### Implications for CSFP

The diagnosis and treatment of CSFP is often overlooked [30]. However, this coronary microvascular disorder subtype appears to be a clinically distinct since it has a similar prevalence in women and men, symptoms often occur at rest, and standard exercise ECG tests are often negative for myocardial ischemia [31]. Randomised controlled clinical trials utilizing calcium channel blockers (diltiazem [32] and mibefradil [9]) have been shown to be effective in preventing the recurrent angina associated with this condition. The latter is particularly worthy of mention, as mibefradil is a novel calcium channel blocker that is particularly effective at inhibiting ET-1-induced constrictor response in human microvessels [33]. Furthermore, mibefradil reduced angina frequency by 55% and nitrate consumption by 59% in patients with CSFP when utilizing the same clinical trial design as the current study [9]. Along with the other pathophysiological attributes of CSFP, as detailed above (see background, a–e), the ET-1 pathway appears to play a key role in the pathogenesis of angina in patients with CSFP. Hence, this coronary microvascular disorder subtype should be targeted for future anti-anginal studies utilizing endothelin blockers.

This pragmatically designed study has important implications for real-world clinical practice, including the following: (a) all recruited patients had readily diagnosed microvascular dysfunction on the basis of the CSFP, as documented on routine diagnostic angiography; (b) patients had refractory angina and were on background anti-anginal therapy, i.e., zibotentan therapy was not used as a de novo medication, so the response may have been more striking; and (c) a patient-related outcome primary endpoint was utilized, rather than a surrogate clinical measure. Accordingly, the study is directly translatable to contemporary clinical practice.

The main limitation of this study is its small sample size, which can be attributed to challenges in identifying patients with CSFP who had frequent angina despite conventional anti-anginal therapy. Recruitment was further impeded by the COVID-19 pandemic and the limited availability of the trial medication. Another limitation is the limited insight into the relative clinical benefits of zibotentan compared to other anti-anginal agents, since the study was not an active-control comparison (e.g., newly diagnosed CSFP patients randomized to zibotentan or diltiazem). Also, the combined use of multiple anti-anginal agents with additive effects may predispose patients to adverse effects. Considering the benefits demonstrated with zibotentan compared to placebo in this study, a large, randomized, active-control, comparative trial design could be justified in the treatment of CSFP.

## 5. Conclusions

In summary, this pilot study has suggested that the administration of 10 mg of zibotentan has a positive impact on reducing angina frequency in patients with refractory angina attributed to the CSFP, underscoring the potential therapeutic efficacy of targeting the ET-1 pathway in this specific disorder. While the primary endpoint, angina frequency, demonstrated significance, secondary endpoints such as angina severity and prolonged angina episodes did not show notable improvements with zibotentan use. Considering its modest efficacy in mitigating angina symptoms and the rate of side effects amongst participants, this underscores the necessity for further investigations to substantiate and validate the therapeutic potential of modulating the ET-1 pathway in the management of CSFP. Further research with a larger cohort and a rigorous methodology is warranted to ascertain the clinical utility of these preliminary findings.

## Figures and Tables

**Table 1:** Baseline characteristics.

Participant characteristics	
Number of patients	18
Age	63.2 ± 9.9 years
Female	33% (6)
Smoking (current)	(0)
Hypertension (on treatment)	56% (10)
Diabetes (on treatment)	11% (2)
Family history of CAD	39% (7)
Hypercholesterolaemia (on treatment)	56% (10)
Concomitant medications	
Aspirin	33% (6)
Statins	67% (12)
Calcium channel blockers	77% (14)
ACE-inhibitors/ARB	28% (5)
Beta blockers	11% (2)
Long-acting nitrates	61% (11)
Angina Diary	
Angina episodes/week (n, SE)	10.6 (2.1)
Total duration/week (mins, SE)	511 (136)
GTN consumption/week (n, SE)	3.9 (1.8)
SAQ components	
Angina frequency	50.0 (50.0, 50.0)
Physical limitations	87.5 (80.6, 100.0)
Treatment satisfaction	96.9 (93.8, 100.0)
Angina severity	
Angina-specific quality of life	50.0 (41.7, 58.3)
SF-36 Components	
Physical Component Summary	44.2 (41.0, 48.7)
Mental Component Summary	52.6 (43.5, 56.5)

ACE: Angiotensin-Converting Enzyme; ARB: Angiotensin Receptor Blockers; CAD: Coronary Artery Disease, SAQ: Seattle Angina Questionnaire; SF-36: Short-Form Health Survey-36; SE: Standard Error.

**Table 2:** Angina diary endpoints.

Study Endpoint	Placebo (n or mins, SD)	Zibotentan (n or mins, SD)	Mean Ratio (95% CI)	*p* Value
Angina episodes/week	10.9 (2.3)	7.5 (2.3)	3.4 (0.1, 6.6)	0.0415
Total duration/week	524.0 (167.0)	503.0 (168.0)	1.35 (0.78, 2.34)	0.7425
GTN consumption/week	3.6 (0.9)	2.4 (0.9)	1.19 (0.31, 2.07)	0.0113

CI: confidence interval; GTN: glyceryl trinitrate; mins: minutes; n: total number; SD: standard deviation.

**Table 3:** Health status instruments.

	Placebo	Zibotentan	Mean Difference (95% CI)	*p* Value
SAQ (Score 0–100; recorded at the end of each phase)				
Angina frequency	54.6 (6.5)	65.4 (6.5)	−10.8 (−31.6, 10.1)	0.6931
Physical limitation	82.8 (4.6)	86.9 (4.6)	−4.2 (−12.0, 3.7)	0.8526
Treatment satisfaction	93.3 (2.0)	97.3 (2.0)	−4.0 (−10.1, 2.2)	0.2890
Angina-specific quality of life	55.4 (5.8)	60.2 (5.8)	−4.8 (−21.6, 11.9)	0.8243
SF-36 Health Questionnaire (Score 0–100; recorded at the end of each phase)				
Physical Component Summary	45.0 (2.1)	42.4 (2.1)	2.63 (−1.33, 6.59)	0.1800
Mental Component Summary	52.4 (2.0)	50.2 (2.0)	2.21 (−1.69, 6.12)	0.2493

CI: confidence interval; SAQ: Seattle Angina Questionnaire; SF-36: Short-Form Health Survey-36.

**Table 4:** Frequency of adverse drug reactions observed for treatments.

	Placebo	Zibotentan	*p* Value
Serious adverse events	0	0	
Adverse drug reactions			
Headaches	0	4	0.10
Ankle swelling	1	10	0.006
Nasal congestion	0	10	0.006
Symptomatic hypotension	0	5	0.04
∆ Heart rate	−3 ± 6	−0.6 ± 12	0.678
∆ Systolic blood pressure	−2 ± 9	−9 ± 16	0.098

## Data Availability

The data presented in this study are available on request from the corresponding author. The data are not publicly available due to privacy and ethical restrictions.
